# Probiotics of *Lacticaseibacillus paracasei* SD1 and *Lacticaseibacillus rhamnosus* SD11 attenuate inflammation and β-cell death in streptozotocin-induced type 1 diabetic mice

**DOI:** 10.1371/journal.pone.0284303

**Published:** 2023-04-11

**Authors:** Jongdee Nopparat, Pissared Khuituan, Saranya Peerakietkhajorn, Rawee Teanpaisan

**Affiliations:** 1 Faculty of Science, Division of Health and Applied Sciences, Prince of Songkla University, Hat Yai, Songkhla, Thailand; 2 Center of Excellence for Trace Analysis and Biosensor, Prince of Songkla University, Hat Yai, Songkhla, Thailand; 3 Faculty of Science, Division of Biological Science, Prince of Songkla University, Hat Yai, Songkhla, Thailand; 4 Faculty of Dentistry, Research Center of Excellence for Oral Health, Prince of Songkla University, Hat Yai, Songkhla, Thailand; Helwan University, EGYPT

## Abstract

Probiotics provide health benefits in various aspects and are believed to modulate the immune system by balancing gut microbiota homeostasis, termed the “microbiota-immune axis”. Recent evidence supports that several *Lactobacillus* strains possess glucose-lowering and anti-inflammatory effects in an animal model of type 1 diabetes (T1D). Although probiotics of *Lacticaseibacillus paracasei* SD1 (SD1) and *Lacticaseibacillus rhamnosus* SD11 (SD11) exert human oral health benefits by reducing harmful bacterial populations, their clinical application regarding hypoglycemic-related traits as well as the underlying mechanisms are still lacking. In this report, we used multiple low doses of streptozotocin (STZ)-induced diabetic BALB/c mice to explore the effects of SD1 and SD11 supplementation on the regulation of markers related to T1D. Experimental mice were randomly assigned into five groups, non-STZ + V, STZ + V, STZ + SD1, STZ + SD11, and STZ + SDM (mixture of SD1 and SD11), and physiological data were measured every week. Blood and pancreas samples were collected at 4- and 8-weeks. Our results indicate that supplementation with SD1, SD11, or SDM for 8 weeks significantly improved body weights, glycemic levels, glucose tolerance, insulin levels, and lipid profiles. Probiotic administration also preserved islet integrity and increased β-cell mass in STZ-injected mice, as well as prevented infiltration of macrophages, CD4^+^, and CD8^+^ T cells into the islets. Significantly, SD1 and SD11 suppressed the levels of IL1-β, TNF-α and IFN-γ and increased IL-10, which is concomitant with the inhibition of cleaved caspase 3, caspase 9, caspase 8, proapoptotic Bax, NF-κBp65, pSTAT1, and iNOS. Additionally, the survival ability of β-cells was mediated by upregulated anti-apoptotic Bcl2. We conclude that SD1 and SD11 attenuate STZ-induced diabetic mice by stabilizing glycemic levels and reducing inflammation, thereby protecting β-cells. Among the probiotic treatment groups, SD11 revealed the best results in almost all parameters, indicating its potential use for alleviating hyperglycemia-associated symptoms.

## Introduction

In type 1 diabetes (T1D), insulin-producing pancreatic β-cells are destroyed by a β-cell-specific autoimmune process that is regulated primarily by cytotoxic T cells [1]. Although extensive effort has been made to decipher all the factors and pathways involved in T1D pathogenesis, exactly where this autoimmune activation starts is not yet fully understood. These episodes are followed by the attraction of other immune cells, such as macrophages, into the islets, resulting in the development of insulitis, causing increased β-cell death and decreased β-cell mass, leading to insulin insufficiency [1]. Consequently, chronic hyperglycemia develops, and T1D patients are dependent on administered insulin for survival [2,3]. Hence, targeting proinflammatory cytokines such as interferons induces gene expression in β-cells that facilitates T-cell-mediated destruction, including upregulation of MHC class I, chemokines, and cell death regulators has been proposed as a potential strategy to overcome this autoimmune destruction. However, the use of immunosuppressors such as anti-CD20 chimeric mAb or humanized anti-CD3 mAbs has failed to show long lasting effects alone, with controversy regarding their dose-efficacy-safety [2]. Therefore, exploring new approaches is required to manage T1D.

Our understanding of the health-promoting beneficial interaction between probiotics and commensal microorganisms has increased substantially in recent years [4,5]. Due to increasing levels of health awareness and consumer consciousness regarding gut health and the concept of preventive health care, the search for new probiotic functional food and dietary supplements is remarkedly growing. Several common underlying mechanisms of probiotics have been proposed, including repairing gut membrane integrity and permeability [6], balancing commensal microbes to affect metabolism, and interfering with infection by pathogens [4]. Additionally, growing clinical evidence supports the role of probiotics in modulating the immune system response in the prevention and treatment of certain diseases, including T1D [5]. Specifically, there are substantial data showing that the gut microbiota interacts closely with the immune system (known as the “gut microbiota-immune axis”) and that perturbations (dysbiosis) in these interactions contribute to the development and progression of T1D [4,5]. Moreover, long exposure to high glucose levels in the circulation of the host body further disturbs the diversity and function of healthy microbiota, which eventually worsens T1D symptoms and vice versa [7]. However, this dysbiosis can be reversed by modifying the gut microbiota through probiotic supplementation, leading to improvement of the host’s metabolic and immune status [8]. Among all probiotic strains, *Lactobacillus* is one of the most commonly used and studied bacteria in humans, and two main mechanisms have been proposed to modulate gut microbiota balance and the host immune system in T1D: 1) by suppressing the innate immune response via Toll-like receptors (TLRs). Specifically, the consumption of selected probiotic strains has been found to decrease the level of proinflammatory cytokines, including IL-6, IL-1β, and TNF-α, while increasing that of anti-inflammatory cytokines, such as IL-10 and TGF-β [9,10] and/or 2), by upregulating FFAR 2/3 via the production of short-chain fatty acids (SCFAs), which could enhance the production of glucagon-like peptide-1 (GLP-1) from intestinal L-cells, thereby lowering blood glucose levels [11]. This evidence highlights the potential of probiotics as a promising intervention to combat autoimmune destruction of β-cells in T1D by restoring homeostasis of the gut microbiota-immune axis, granting host health benefits.

*Lacticaseibacillus paracasei* SD1 (SD1) and *Lacticaseibacillus rhamnosus* SD11 (SD11) were first isolated and characterized from caries-free children [12,13]. Previous clinical trials investigated their effect on oral microflora in human volunteers and revealed their strong inhibitory effect against *Streptococcus mutans* and *Streptococcus sobrinus*, as well as gram-negative periodontal pathogens such as *Porphyromonas gingivalis* and *Aggregatibacter actinomycetemcomitans*, without adverse effects even after long-term administration (milk powder containing SD1 or SD11 once daily for up to six months) [14–17]. These clinical trial studies ensure their oral care benefits and safety for oral consumption in humans; hence, the main objective of our present work was to further document that probiotics of the SD1 and SD11 strains could potentially intervene in the hyperglycemia-induced inflammatory destructive environment, thereby improving β-cell survival. To address these unexplored areas, we treated a mouse model with multiple low doses (MLD) of STZ-induced T1D with the probiotics SD1, SD11, or a mixture of SD1 and SD11, designated SDM, for 4 and 8 weeks. We previously established an animal model by injecting 50 mg/kg STZ for 5 consecutive days [18]. This optimized approach is designed to induce partial damage to pancreatic β-cells, leading to initiation of immune cell homing into the islets and triggering an inflammatory process, thereby mimicking T1D pathogenesis. We performed each of the experiments according to subsequent immune response events causing the loss of β-cell mass through an apoptotic pathway. Physiological changes, feeding behaviors, serum biochemistry, pancreas histopathology, intracellular immune cells, proinflammatory cytokines and apoptotic markers were observed. This study will facilitate the development of probiotic-based interventions to ameliorate T1D-associated symptoms.

## Materials and methods

### Preparation of *Lacticaseibacillus paracasei* SD1 and *Lacticaseibacillus rhamnosus* SD11

Two probiotic strains, SD1 and SD11, were used in this study. The identifications, culture conditions, and probiotic preparations were described previously [12,13]. In the present work, the probiotic strains were recovered from storage at -80°C on de Man, Rogosa and Sharpe (MRS) agar plates and inoculated into 50 mL of MRS broth overnight under anaerobic conditions (80% N_2_, 10% H_2_ and 10% CO_2_) at 37°C. The cultures were then added to 450 mL of MRS broth and kept under anaerobic conditions at 37°C for 48 h. Cells were harvested by centrifugation (3,000 × *g*, 5 min) from the MRS broth and washed 3 times with 0.85% NaCl before being used. For feeding purposes via gavage, probiotics were freshly prepared by being dissolved in distilled water to reach a final concentration of 10^9^ colony-forming units (CFU)/200 μl/mouse/day. This effective concentration was selected in accordance with previous clinical studies [14–17].

### Animal and induction of diabetes

**Fig 1:**
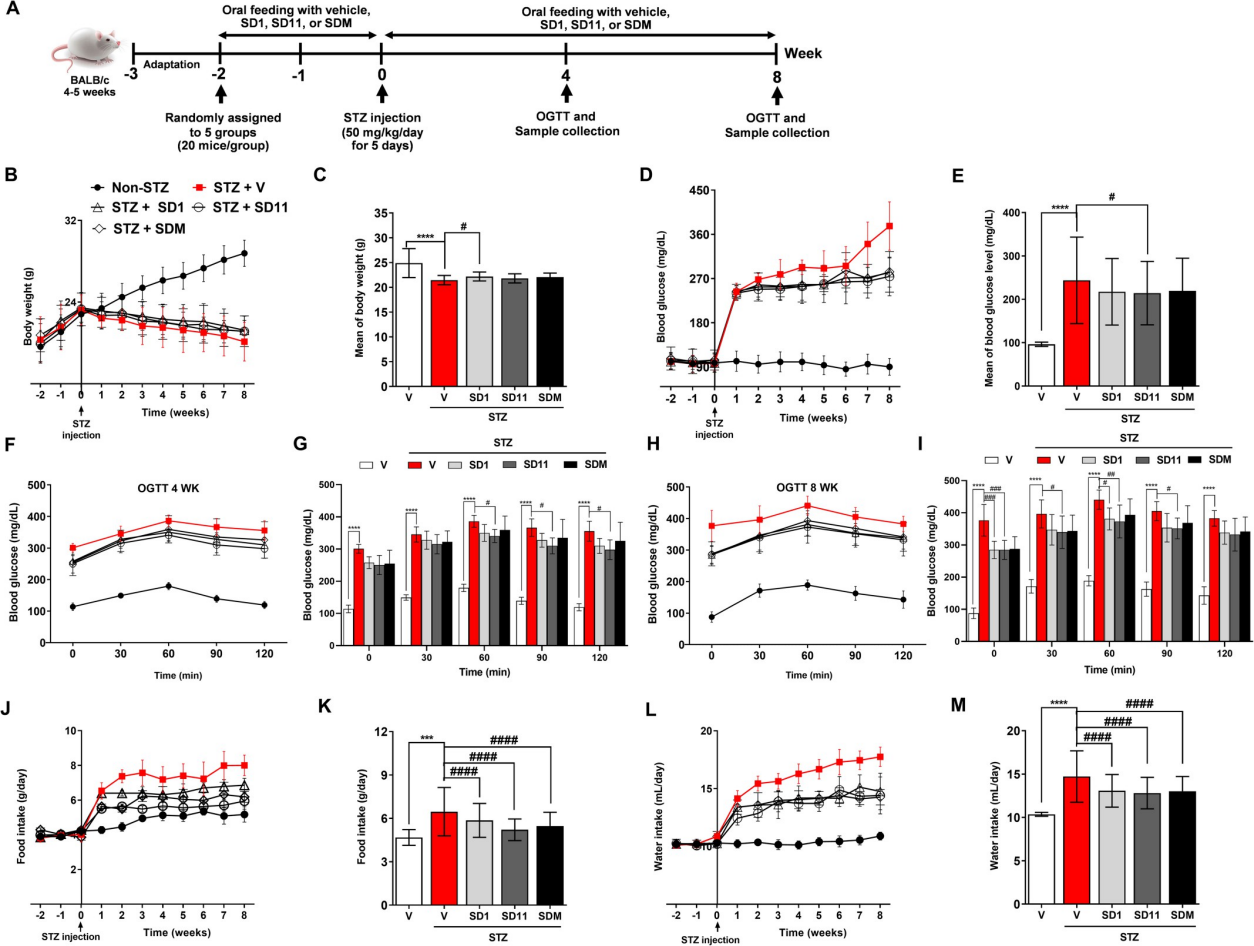
Probiotics ameliorate physiological and feeding behavior changes in STZ-mice. (A) Schema of the study timeline, (B) and (C) changes in body weights, (D) and (E) fasting blood glucose levels, (F) and (G) oral glucose tolerance test at 4 weeks, (H) and (I) oral glucose tolerance test at 8 weeks, (J) and (K) food intake, and (L) and (M) water intake of the experimental mice throughout the entire study (10 weeks). Values are the mean ± S.D. (n = 10 and n = 8 per group for the 4- and 8-week time points, respectively). ****P < 0.0001, ***P < 0.001 vs. the corresponding non-STZ group; ^####^*P* < 0.0001, ^###^*P* < 0.001, ^##^*P* < 0.01, ^#^*P* < 0.05 vs. the corresponding vehicle-treated STZ group.

Animal care and procedures were approved by the Institutional Animal Care and Use Committee of Prince of Songkla University (MHESI 68014/2307, Ref. 104/2021). The animals were purchased from Nomura Siam International Co., Ltd., based in Bangkok, Thailand and were maintained under standard conditions of temperature (25 ± 2°C) and humidity (50 ± 10%) with an alternating 12-h light/dark cycles in the laboratory animal service center of Prince of Songkla University. As depicted in Fig 1A, all animals were acclimatized under laboratory conditions for one week prior to experiments. Diabetes was induced intraperitoneally for 5 consecutive days with either 50 mg/kg of STZ (freshly prepared by dissolving in 0.1 M citrate buffer, pH 4.5) or citrate buffer alone (normal control animals) [18]. Week 0 was defined as the first day of STZ injection. 72 h after the last STZ injection, fasting blood glucose (FBG) level from the tail vein was examined using a blood glucose meter (Accu-Check Active ® and test strips, Roche diagnostic, Mannheim, Germany). Mice were considered diabetic when blood glucose levels were above 200 mg/dL [18–20].

### Experimental design

The determination of the sample size was calculated by MINITAB statistical analysis package (Minitab 18, Minitab Inc., Pennsylvania, USA) in accordance with the method of Ko and Lim [21]. In this experiment, 100 male BALB/c mice (4–5 weeks old) weighing approximately 17–21 g were used at the beginning of the experiment. After a 1-week adaptation period, mice were randomly assigned to 5 treatment groups of 20 mice as follows (Fig 1A):

Group 1 non-STZ + V, normal control mice were fed distilled water (vehicle, V)

Group 2 STZ + V, diabetic control mice were fed distilled water.

Group 3 STZ + SD1 served as diabetic mice given 10^9^ CFU/mL SD1.

Group 4 STZ + SD11 served as diabetic mice given 10^9^ CFU/mL SD11.

Group 5 STZ + SDM served as diabetic mice given a 10^9^ CFU/mL of SD1 and SD11 mixed together.

To minimize the risks of bias, the present study was performed in a single-blind fashion. Normal control and diabetic control mice received equal volumes of distilled water in place of the probiotics daily. Mice continued to be fed vehicle, SD1, SD11 or SDM by gavage once daily for 4 weeks or 8 weeks. Body weights (BW) and FBG levels were observed weekly. Food consumption and water intake were measured daily before administration. After the 4- and 8-week time points, no losses of animals were observed and they were euthanized with thiopental (70 mg/kg b.wt.) to collect blood and the pancreas for further experiments. Of note, for Western blot analysis, protein samples from each experimental group cannot be omitted to the examiners because the samples must be loaded into the gels in a particular pattern for representative and comparative purposes.

### Oral glucose tolerance test (OGTT)

The OGTT test was conducted a day before euthanization at the 4- and 8-week time points by oral administration of 1 ml glucose (1 g/kg) (n = 10 and n = 8 per group for the 4- and 8-week time points, respectively). The blood glucose levels were then analyzed at 30 min, 60 min, 90 min, and 120 min after administration using a blood glucose meter (Accu-Check Active ® and test strips).

### Serum biochemical analysis

Assessment of serum triglycerides (TG), total cholesterol (TC), low-density lipoprotein cholesterol (LDL-c), high-density lipoprotein cholesterol (HDL-c), and free fatty acids (FFA) was measured using commercial assay kits (Cell Biolabs, Inc., San Diego, CA, USA). Briefly, biochemical markers were performed by collecting blood samples from overnight fasting (12 h) mice (n = 6 per group from both time points) from cardiac puncture and then allowing the blood to clot at room temperature (RT) for 30 min. Serum was collected by centrifugation at 2,000 x *g* at 4°C for 30 min and kept at -80°C until use. The serum was diluted before assaying (1:100 to 1:200 with assay diluent), and the absorbance was recorded in the 540–570 nm range on a microplate reader (BioTek Synergy HT Microplate Reader). Then the concentrations of TG, TC, LDL-c, HDL-c, and FFA within samples were calculated by comparing the sample absorbance values to the standard curve.

In addition, the fasting blood insulin (FINS) concentrations were estimated using the Mouse Insulin ELISA Kit (Thermo Fisher Scientific) according to the manufacturer’s protocol. Briefly, ninety-six‐well ELISA plates were loaded with 100 μl of diluted samples and the capture antibody overnight at 4°C with gentle shaking. Following three washes with PBS containing 0.05% Tween-20 (PBST), the wells were coated with biotin conjugate for 1 h at RT. Bound complexes were detected by incubation with streptavidin-HRP solution for 45 min at RT. Following three washes with PBST, 3,3′,5,5′‐tetramethylbenzidine (TMB) was added for 30 min at RT in the dark with gentle shaking. The reaction was stopped by adding 1M H_2_SO_4_ and analyzed using a microplate ELISA reader at 450 nm. The concentrations within the samples were calculated by comparing the absorbance values to the standard curve. The Homeostasis Model Assessment-Insulin Resistance (HOMA-IR) was calculated as follows: HOMA-IR = blood glucose (mmol/L) x insulin (mU/L)/22.5 [22].

### Histological examination of the pancreas

The pancreas was washed with ice-cold PBS and divided into 2 parts, either fixed in 10% buffered formalin for histological examination or processed for Western blot analysis. For histological analysis, tissue samples were processed for dehydration, clearing, and embedding in paraffin. Briefly, paraffin sections (5 μm thick) were placed onto glass slides, deparaffinized in xylene, hydrated through graded ethanol to distilled water, and finally stained with H&E. Morphometric evaluation of the pancreas (n = 6 sections/animal with a 15 μm interval between each section from 6 animals/group) was determined quantitatively by light microscopy in a blinded fashion in regard to (a) the relative value of the islet size, calculated by dividing the sum total islet area by the pancreas sectional area multiplied by 100; and (b) the number of islets per pancreas section determined by dividing the sum total number of islets by the pancreas sectional area multiplied by 100 [18].

### Immunofluorescent staining

For fluorescence microscopy imaging of guinea pig anti-insulin (Abcam, Cambrdige, UK), rabbit anti-iNOS (Cell Signaling Technology, Inc) and rabbit anti-cleaved-caspase 3 (Cell Signaling), paraffin-embedded tissue sections were deparaffinized in xylene, hydrated through graded ethanol to distilled water, and permeabilized in PBS with 0.1% Triton X-100 (PBST) for 10 min. Blocking was performed using 5% BSA in PBST for 1 h at RT, followed by incubation with antibodies diluted 1:100 in 1% BSA in PBST at 4°C overnight. Three consecutive washes with PBST for 5 min each were followed by sequential incubation with either Alexa Fluor 488 goat anti-guinea pig or Alexa Fluor 594 goat anti-rabbit secondary antibodies (Invitrogen) diluted 1:300 in 1% BSA in PBST at RT for 1 h. Then, the slides were washed 3 times with PBST and mounted using prolonged diamond anti-fade mounting medium containing 4’,6-diamidino-2-phenylindole (DAPI) (Invitrogen). Images were captured under a fluorescence microscope (Olympus D73 equipped with CellSens software). Quantifications of the percentage of islets containing insulin, apoptotic β-cells or iNOS were performed by the manually counted number of insulin, cleaved-caspase 3, and iNOS positive staining cells divided by the islet area multiplied by 100. A total of 50 islets per experimental group were counted in a blinded fashion: 5 islets per section, 2 sections per mouse with a 15-μm interval between each section, and 5 mice per group.

### Immunohistochemical analysis

IHC was conducted according to the manufacturer’s instructions (Vectastain Elite ABC-HRP kit; Vector Laboratories, Inc.; Maravai Life Sciences). The following antibodies were utilized: rabbit anti-F4/80^+^, rabbit anti-CD4^+^, rabbit anti-CD8^+^, rabbit anti-IL-1β, rabbit anti-TNF-α (Cell signaling), and rabbit anti-IFN-γ (Abcam). Briefly, pancreas tissue sections were deparaffinized with xylene and rehydrated in a graded ethanol series. Antigen retrieval was performed by heating the tissues at 95°C with citrate buffer (pH 6.0) for 20 min, followed by blocking endogenous peroxidase activity in 0.3% H_2_O_2_ (in methanol) for 30 min at RT. Following three 5 min washes with PBS with 0.1% Triton X-100 (PBST), normal horse serum (Vector Laboratories) was applied for 1 h at RT. Incubation with primary antibodies was performed overnight at 4°C in a humidified chamber. The tissues were then rinsed with PBST and incubated with a biotinylated horse anti-mouse/rabbit immunoglobulin G universal secondary antibody (Vector Laboratories) for 1 h at RT. After washing with PBST, the tissues were stained with diaminobenzidine (DAB), rinsed with distilled water and counterstained with hematoxylin for 1 min at RT. The sections were then dehydrated in a series of graded ethanol solutions, cleared in xylene, and mounted for light microscopic examination. Digital images were randomly selected, and infiltration of immune cells in the STZ-hyperglycemic models was quantified by counting the number of positively stained cells per islet in the same manner as fluorescent image analysis.

### Western blot analysis

Additional antibodies were obtained from the following sources: rabbit anti-IL-10, rabbit anti-caspase 8, mouse anti-caspase 9, rabbit anti-NF-κBp65, rabbit anti-pSTAT1 (Cell signaling), and rabbit-β-actin (Abcam). Pancreatic lysates from 3 mice from each group were prepared in ice-cold RIPA buffer (Sigma-Aldrich) supplemented with 1x protease inhibitor cocktail (Invitrogen). Tissue lysates were then cleared by centrifugation at 14,000 x *g* for 30 min at 4°C. The total protein concentration was determined by a BCA protein assay kit (Thermo Scientific). Approximately 40 μg of protein from each sample was separated by 10–12% SDS/PAGE, and the proteins were transferred to PVDF membranes (Merk-Millipore). After being blocked in blocking buffer (5% nonfat dry milk) in TBS containing 0.05% Tween 20; TBST) for 1.5 h at RT, membranes were probed with the primary antibodies overnight in blocking buffer (1: 1,000). After washing 3 times, the membranes were incubated for 1 h with HRP-linked secondary antibodies (Sigma-Aldrich) in blocking buffer (1: 5,000). The level of β-actin was estimated in samples to check for equal loading of samples followed by washing 3 times with TBST. Finally, antigen-antibody complexes were observed using Luminata Crescendo Western HRP substrate according to the manufacturer’s instructions (Merk-Millipore), and pictures were captured by a gel document system for chemiluminescence (Alliance Q9-Atom, UVITEC Cambridge). The density of the immunoreactive bands was analyzed using NIH ImageJ software. Data were normalized to the loading control and are expressed as the fold change compared to the normal control group, and then statistical analysis was conducted.

### Statistical analysis

The results are shown as means ± standard error of the mean (SEM) unless noted otherwise. Statistical analyses were performed using one-way or two-way analysis of variance (ANOVA) followed by Tukey’s multiple comparisons with the GraphPad Prism (GraphPad Software version 8.0.1, San Diego, CA) and considered significant at *P* < 0.05.

## Results

### Probiotics reduce STZ-induced physiological and physical abnormalities associated with hyperglycemia

First and foremost, as shown in Fig 1A, we documented that the probiotics given by gavage orally 2 weeks prior to diabetic induction were safe and did not result in diarrhea, mortality, or toxic effects on the behavior of the treated mice. In addition, the probiotics did not affect the physiological parameters, including BW, FBG, food and water intake, among the groups. Then, we induced hyperglycemia via STZ injection, which is one of the most applicable chemicals for inducing diabetes. After STZ induction, we continued to monitor the physiological and physical parameters for 8 weeks. Diabetes-related symptoms developed after one week of STZ administration, and the changes were pronounced toward the last two weeks of the experimental period. Although the probiotics could not completely prevent the decline in BW (Fig 1B and 1C), SD1-treated mice showed significantly improved BW in comparison to vehicle-treated STZ-animals (Fig 1B and 1C), while SD11-treated mice were able to maintain lower FBG levels throughout the experiment, resulting in a significantly lower FBG when compared to that of vehicle-treated STZ mice (Fig 1D and 1E). Meanwhile, the OGTT test also confirmed that STZ disrupted the glucose metabolism capacity in vehicle-treated STZ mice when compared to the non-STZ-group (Fig 1F–1I). Supplementation with SDM resulted in significantly lower glucose values at 60, 90, and 120 min and at 30, 60, and 90 min after glucose overload at the 4-week (Fig 1F and 1G) and 8-week (Fig 1H and 1I) time points, respectively, while SD1-treated mice had significantly decreased BG at 60 and 120 min. Food consumption (Fig 1J and 1K) and water intake (Fig 1L and 1M) observations showed that vehicle-treated STZ mice consumed significantly more food and water than those in the non-STZ group, which are common signs of diabetes. In contrast, all probiotics resulted in a significant reduction in these parameters throughout the experimental period (Fig 1J–1M).

### Probiotics improve serum biochemistry in STZ-treated mice

FINS levels were monitored at weeks 4 and 8 in all groups. As depicted in Table 1, FINS levels in the vehicle-treated STZ group were remarkably lower than those in the non-STZ group at both time points, while FINS in the SD1-supplemented groups at 8 weeks increased significantly. However, supplementation with SD1, SD11, or SDM for 4 or 8 weeks had no impact on the HOMA-IR index.

**Table 1:** Assessment of serum biochemistry among experimental groups.

Group	Week	FINS (μg/L)	HOMA-IR	TG (mg/dL)	TC (mg/dL)	LDL-c (mg/dL)	HDL-c (mg/dL)	FFA (μg/mL)
**Non-STZ + V**	4	2.23 ± 0.47	0.63 ± 0.16	82.40 ± 10.20	86.20 ± 12.43	63.24 ± 12.45	6.48 ± 1.19	42.23 ± 2.52
	8	2.36 ± 0.33	0.53 ± 0.15	89.00 ± 10.34	97.33 ± 13.67	72.63 ± 12.00	6.90 ± 0.59	41.45 ± 1.68
**STZ + V**	4	1.17 ± 0.19***	0.81 ± 0.13	195.80 ± 14.77****	175.00 ± 25.42****	131.84 ± 25.2****	4.00 ± 0.65***	48.47 ± 2.09***
	8	0.89 ± 0.07****	0.69 ± 0.09	206.50 ± 24.48****	190.67 ± 29.48****	145.27 ± 27.01***	4.10 ± 0.99****	49.23 ± 2.53**
**STZ + SD1**	4	1.57 ± 0.31	0.90 ± 0.21	148.00 ± 22.37^##^	129.40 ± 13.92^###^	94.56 ± 10.9^#^	5.24 ± 0.77	45.73 ± 2.65
	8	1.34 ± 0.20*	0.75 ± 0.09	168.50 ± 29.63	119.00 ± 15.60	80.02 ± 11.72^##^	5.28 ± 0.68	45.66 ± 3.09
**STZ + SD11**	4	1.52 ± 0.40	0.86 ± 0.20	144.60 ± 31.33^##^	136.40 ± 10.98^##^	103.24 ± 15.26	4.24 ± 1.00	44.52 ±1.93
	8	1.38 ± 0.26	0.81 ± 0.18	148.83 ± 21.20^##^	124.67 ± 26.35^##^	88.90 ± 27.14^##^	6.00 ± 0.58^##^	45.83 ± 3.17
**STZ + SDM**	4	1.53 ± 0.32	0.89 ± 0.18	149.4 ± 16.83^##^	151.80 ± 24.16^##^	117.74 ± 24.8	4.18 ± 0.79	46.71 ± 2.56
	8	1.34 ± 0.28	0.80 ± 0.16	156.00 ± 26.11^#^	131.17 ± 30.12^##^	94.32 ± 33.26^#^	4.98 ± 0.94	43.33 ± 2.08

FINS, Fasting insulin; HOMA-IR, Homeostasis model assessment-insulin resistance TC, Total cholesterol; TG, Triglyceride; LDL-c, low-density lipoprotein cholesterol; HDL-c, high-density lipoprotein cholesterol; FFA, free fatty acid.

Values represent the means ± SD (n = 6 per group).

****P < 0.0001

***P < 0.001

**P < 0.01 vs. the corresponding non-STZ group.

^*###*^*P* < 0.001

^*##*^*P* < 0.01

^*#*^*P* < 0.05 vs. the corresponding vehicle treated-STZ group.

Hyperglycemia can also cause lipid metabolism dysfunction [20]. Hence, we further determined the effect of probiotics on serum levels of TC, TG, LDL-c, HDL-c, and FFAs. As expected, compared with non-STZ mice, all the parameters were significantly elevated in the vehicle-treated STZ group, while the HDL-c levels decreased significantly at both time points. We observed that each probiotic induced beneficial effects on the abnormal lipid profiles in STZ-induced diabetic mice to a certain degree. At 4 weeks, SD1-, SD11- and SDM-treated mice had significantly reduced TG and TC levels, but only the SD1-treated group had significantly improved LDL-c levels. At 8 weeks, SD1 treatment significantly reduced only LDL-c levels; in contrast, SD11 and SDM treatments strongly decreased TC, TG, and LDL-c levels. Moreover, a significant reduction in HDL-c was detected with SD11 supplementation for 8 weeks. However, no significant difference in FFAs was detected in probiotic-supplemented mice compared to untreated STZ mice.

### Probiotics preserve islet structure and β-cell mass in STZ-treated mice

**Fig 2:**
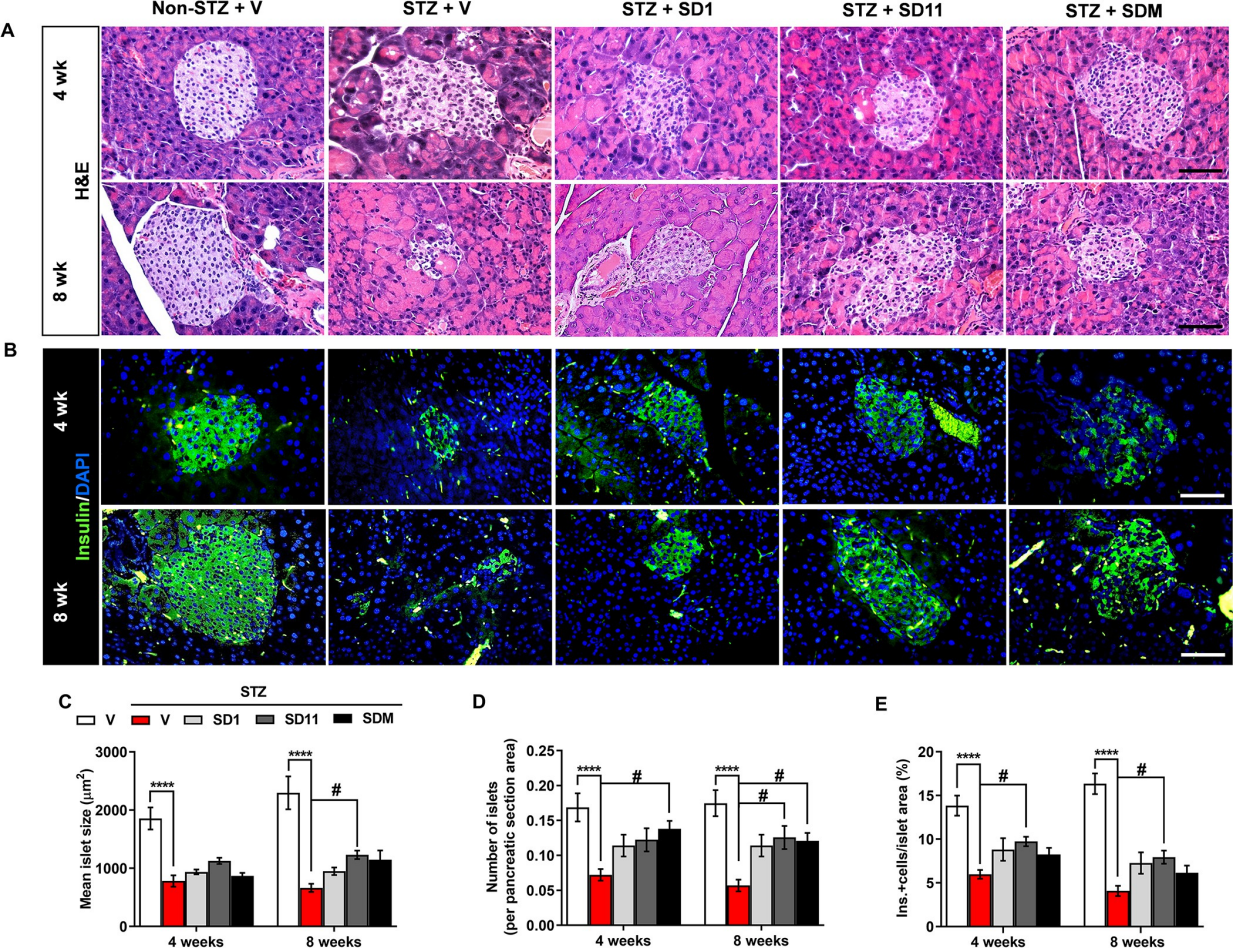
Probiotics attenuate STZ-induced histopathological changes in the pancreases of mice. Representative images of the pancreatic tissues from different groups were examined using (A) H&E staining and (B) β-cell marker insulin (green) and DAPI (blue) staining at the 4- and 8-week time points. (C) Distribution of islet sizes among experimental groups. (D) The average number of islets per cross-sectional area of all islets found in each group. (E) Percentage of insulin-positive cells per islet area. All images were taken at 400 magnifications. Scale bars, 50 μm. Values represent means ± SEM. Data were obtained from 36 histological stained sections per 6 mice per group for H&E. A measurement of 50 independent islets per group (10 islets per mouse pancreas were randomly chosen from 5 animals per group) were examined for immunofluorescent staining. ****P < 0.0001 vs. the corresponding non-STZ group; ^#^*P* < 0.05 vs. the corresponding vehicle-treated STZ group.

Generally, STZ is selectively taken up by pancreatic β-cells, as reflected by pathological changes in the diabetic pancreas and hypoglycemic conditions. By H&E (Fig 2A) and immunofluorescence (Fig 2B) examinations, compared with the non-STZ group, islets in the vehicle-treated STZ group showed obvious endogenous destruction, ill-defined borders, and cell rupture at both time points. In addition, notably reduced and atrophied islets (Fig 2C) containing significantly fewer islets (Fig 2D) and β-cells (Fig 2E) were observed in the diabetic pancreas. Although the prevention of pathological changes in probiotic-treated pancreases was not complete, probiotics potentially prevented the above abnormalities (Fig 2A–2E). We found that at 8 weeks, supplementation with SD11 was attributed to a significant increase in the size (Fig 2C) and number (Fig 2D) of islets, whereas SDM treatment greatly prevented the reduction in size at both time points (Fig 2D). Moreover, SD11-treated mice also notably increased β-cell mass, as detected by the number of insulin-positive cells at all time points (Fig 2E).

### Probiotics reduce intraislet immune cell infiltration in STZ-treated mice

**Fig 3:**
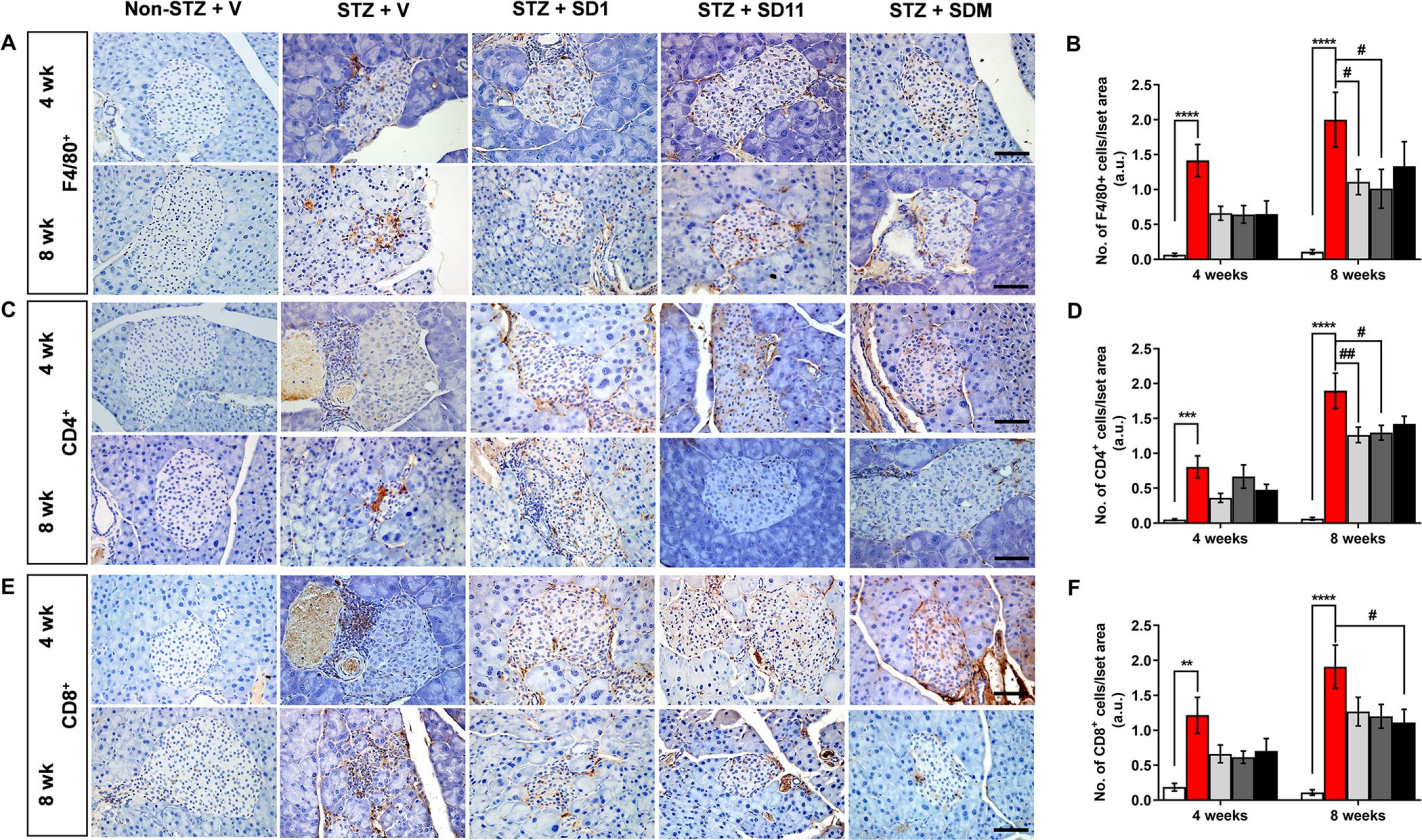
Probiotics attenuate intraislet immune cell infiltration in STZ-treated mice. Representative images and quantification of pancreas sections immunostained for (A-B) the macrophage marker F4/80^+^, the T-cell markers (C-D) CD4^+^, and (E-F) CD8^+^ at the 4- and 8-week time points. All images were taken at 400 magnifications. Scale bars, 50 μm. Values represent means ± SEM. A measurement of approximately 50 independent islets per group (10 islets per mouse pancreas were randomly chosen from 5 animals per group) were examined. ****P < 0.0001, ***P < 0.001, **P < 0.01 vs. the corresponding non-STZ group; ^##^*P* < 0.01, ^#^*P* < 0.05 vs. the corresponding vehicle-treated STZ group.

The cascade of events leading to β-cell apoptosis in T1D begins with the migration of macrophages and dendritic cells to pancreatic islets and their subsequent presentation of β-cell-specific antigens, which induces the differentiation of T cells [5]. Therefore, we next determined whether the beneficial effect of probiotics was associated with attenuation of pancreatic immune cell infiltration (Fig 3). Compared to non-STZ mice, islet macrophage (F4/80^+^) (Fig 3A and 3B), CD4^+^ (Fig 3C and 3D), and CD8^+^ (Fig 3E and 3F) populations were significantly detected in vehicle-treated STZ mice at all time points, with further strengthening at the 8-week time point. Although the difference did not reach a level of significance, treatment with all probiotics for 4 weeks had the potential to reduce the intraislet number of macrophages, CD4^+^, and CD8^+^ T cells (Fig 3A–3F). Moreover, at 8 weeks, SD1- and SD11-treated mice exhibited a significant reduction in macrophages (Fig 3A and 3B) and CD4^+^ T cells (Fig 3C and 3D), whereas the number of CD8^+^ T cells was significantly lower in SDM-treated mice (Fig 3E and 3F) than in vehicle-treated STZ mice.

### Probiotics reduce pancreatic pro-inflammatory cytokines in STZ- treated mice

**Fig 4:**
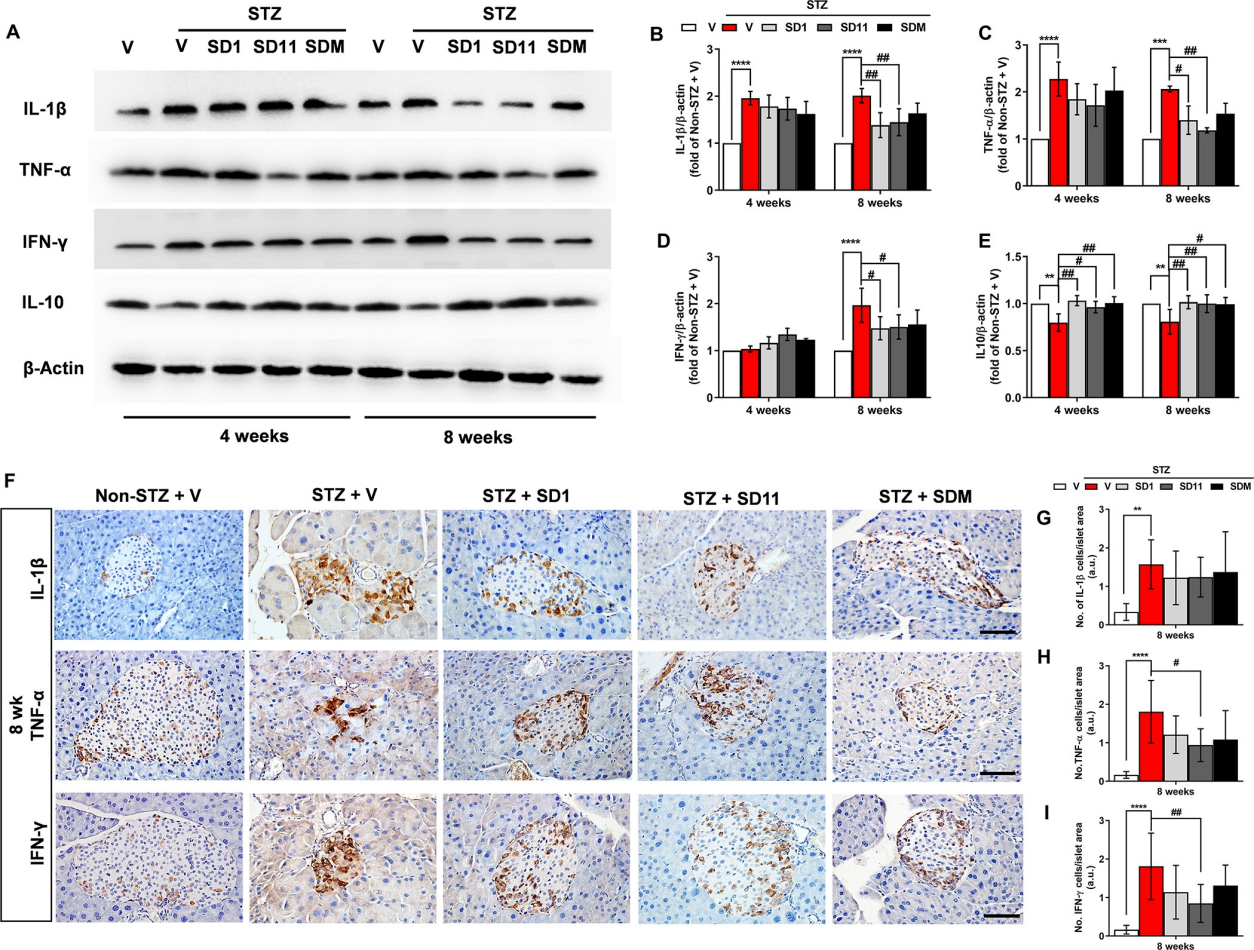
Probiotics attenuate MLDS-induced cytokine expression in the pancreas of mice. (A) Representative images of Western blot analysis of pancreatic cytokines IL-1β, TNF-α, IFN-γ, and anti-inflammatory IL-10. Densitometry quantification of (B) IL-1β, (C) TNF-α, (D) IFN-γ, and (E) IL-10 corrected by β-actin. (F) Representative images of the pancreatic tissues from different groups depict the localizations of IL-1β, TNF-α, and IFN-γ in the islets by immunohistochemical staining, at 400 magnification. Scale bars, 50 μm. Quantification of (G) IL-1β, (H) TNF-α, and (I) IFN-γ. Data are presented as means ± SEM. N = 3 per group for Western blot. A measurement of approximately 50 independent islets per group (10 islets per mouse pancreas were randomly chosen from 5 animals per group) were examined for immunostaining. ****P < 0.0001, **P < 0.01 vs. the corresponding non-STZ group; ^##^*P* < 0.01, ^#^*P* < 0.05 vs. the corresponding vehicle-treated STZ group.

Once CD4^+^ and CD8^+^ T cells are stimulated, these cells then secrete the cytokines IL-1β, TNF-α and IFN-γ, which induce the migration of CD8+ cytotoxic T cells to the islets, activating a final common pathway of β-cell apoptosis through the NF-κB and Fas pathways, resulting in progressive β-cell loss in diabetes [9]. Therefore, we then analyzed the changes in cytokine-mediated inflammatory responses by Western blotting assay (Fig 4A–4E) and immunohistochemistry (Fig 4F–4l). We found that diabetes induced strong increases in pancreatic IL-1β and TNF-α, but not IFN-γ, at the protein level at the 4-week time point, and all cytokines were further elevated at the 8-week time point (Fig 4A–4D). Despite a tendency to decrease IL-1β, TNF-α, and IFN-γ levels in probiotic treatments for 4 weeks, significant changes were not detected when compared to vehicle-treated STZ-mice (Fig 4A–4D). However, treatment with SD1 and SD11 significantly attenuated diabetes-induced cytokine levels after treatment for 8 weeks. Additionally, the protein levels of IL-10, a cytokine synthesis inhibitory factor, were tested to confirm the suppressive effect of probiotics on inflammatory cytokines (Fig 4A and 4E). All probiotic treatments markedly increased pancreatic IL-10 levels at both 4- and 8-week compared to vehicle-treated STZ pancreases. Immunohistochemical staining confirmed our findings that IL-1β, TNF-α, and IFN-γ expressions were elevated in diabetic pancreases (Fig 4F–4I). Cytokine-positive cells were found predominantly around the borders of islets and much less frequently in the islet center in all groups, except for vehicle-treated STZ pancreases, in which all islet cells intensely expressed the cytokines IL-1β, TNF-α, and IFN-γ (Fig 4F–4I), concomitant with Western blot results at 8 weeks. Our findings suggest that the administration of probiotics potentially protected against β-cell destruction, at least through the suppression of proinflammatory cytokine production in the pancreas of STZ-induced diabetic mice.

### Probiotics prevent STZ-induced β-cell apoptosis in mice

**Fig 5:**
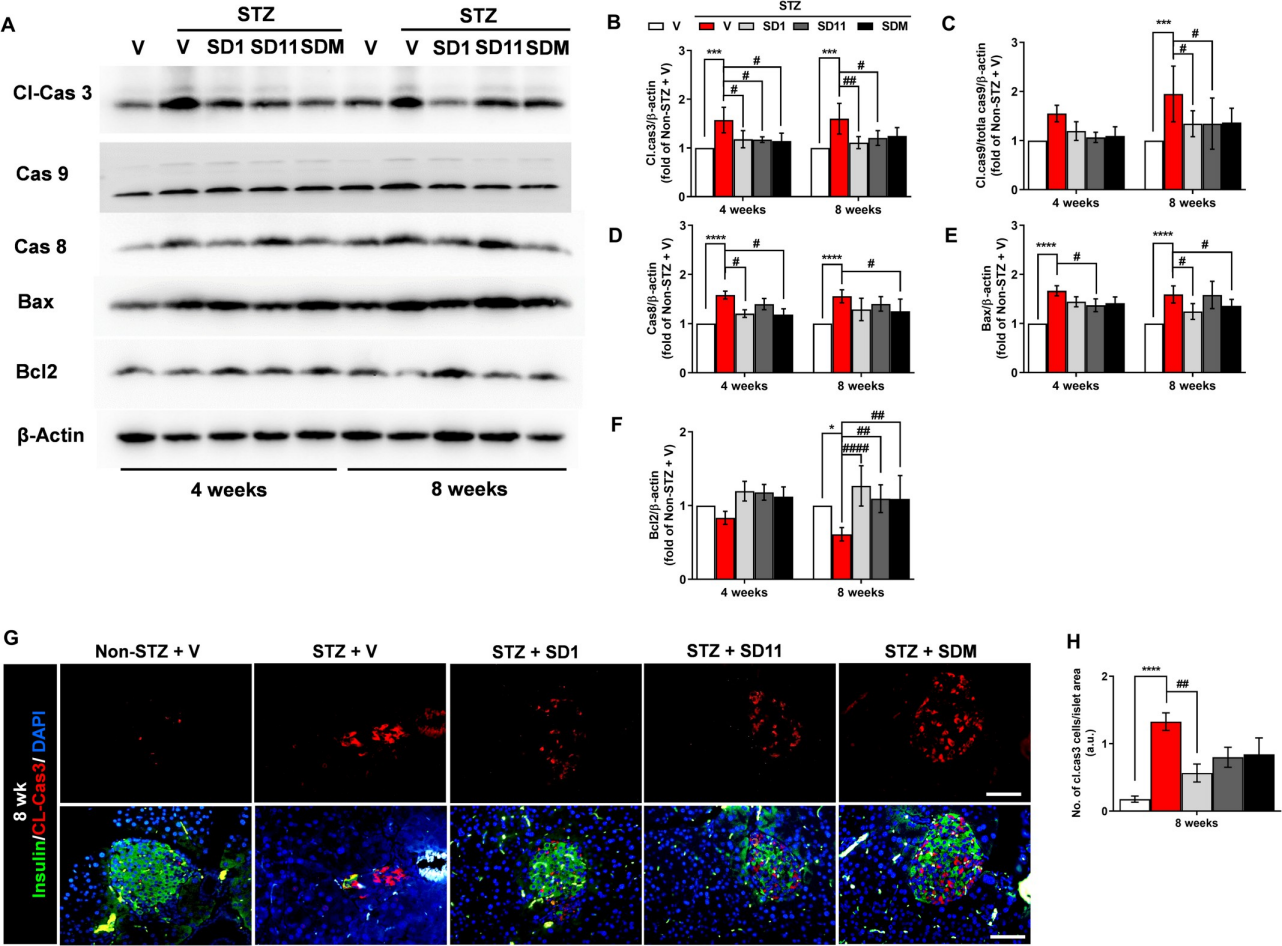
Probiotics preserve β-cells by inhibiting the apoptotic pathway in STZ mice. (A) Representative images of pancreatic protein expression of cleaved caspase 3, total caspase 9 and cleaved caspase 9, caspase 8, Bax, and Bcl2 assessed by Western blotting. Densitometry quantification of (B) cleaved caspase 3, (C) cleaved caspase 9/total caspase 9, (D) caspase 8, (E) Bax, and (F) Bcl2 corrected by β-actin. (F) Representative immunofluorescent images of islets by cleaved caspase 3 (red), β-cell marker insulin (green) and DAPI (blue) staining in sections of formalin-fixed paraffin-embedded among experimental groups at 8-week time point. (G) Quantification of the number of cleaved caspase 3-positive cells per islet area. All images were taken at 400 magnification. Scale bars, 50 μm. Data represent the mean ± SEM. N = 3 for western blot analysis. A measurement of 50 independent islets per group (10 islets per mouse pancreas were randomly chosen from 5 animals per group) were examined for immunostaining. ****P < 0.0001, ***P < 0.001vs. the corresponding non-STZ group; ^####^*P* < 0.0001, ^###^*P* < 0.001, ^##^*P* < 0.01, ^#^*P* < 0.05 vs. the corresponding vehicle-treated STZ group.

STZ preferably targets β-cells, and with MLDS induction, the presence of cytokines and infiltrated immune cells continuously provokes cell death regulators and their downstream proteins, which in turn aggravate β-cell death through apoptosis [23,24]. Hence, we identified several apoptotic proteins to functionally evaluate target molecules in diabetic pancreases with and without probiotic treatment (Fig 5A–5H). Compared with the non-STZ group, vehicle-treated STZ mice displayed a significant upregulation of cleaved caspase 3 (Fig 5A and 5B), caspase 8 (Fig 5A and 5D), and Bax (Fig 5A and 5E) at both time points, whereas cleaved caspase-9 levels were elevated significantly at 8 weeks only (Fig 5A and 5C). Pancreatic cleaved caspase-3 levels were significantly attenuated by SD1, SD11, and SDM at 4 weeks, but only the protective effect of SD1 and SD11 extended to 8 weeks (Fig 5A and 5B). Meanwhile, a significant decrease in cleaved caspase 9 was detected in SD1- and SD11-treated mice at the 8-week time point; in contrast, a similar beneficial effect was not significantly different at the 4-week time point (Fig 5A and 5C). Furthermore, treatment with SD1 showed a significant downregulation of caspase 8 at the 4-week time point, whereas SDM supplementation showed a preventive effect on caspase 8 continuously until the 8-week time-point (Fig 5A and 5D). Likewise, we found that not all probiotic treatments had a beneficial effect on the Bax protein level, in which a significant reduction was found only in the SD11 treatment for 4 weeks and the SD1 and SDM treatments for 8 weeks (Fig 5A and 5E). Further examination of the protein level of Bcl2, an anti-apoptotic molecule (Fig 5A and 5F), showed that STZ stimulation significantly decreased Bcl2 production in the pancreas, whereas all probiotics remarkably preserved Bcl2 production when compared to vehicle-treated STZ mice.

Accordingly, by immunofluorescent staining, untreated STZ mice displayed a noticeable increase in apoptotic β-cells by active caspase-3-positive staining cells (Fig 5G and 5H), concomitant with a significant reduction in insulin-positive staining cells at 8-week time points compared to that of non-STZ mice (Fig 5H). In contrast, a significant reduction in β-cell apoptosis was detected in SD1-treated mice compared to vehicle-treated mice (Fig 5H).

### Probiotics improve the execution of molecules in cytokine-induced β-cell apoptosis in STZ-treated mice

**Fig 6:**
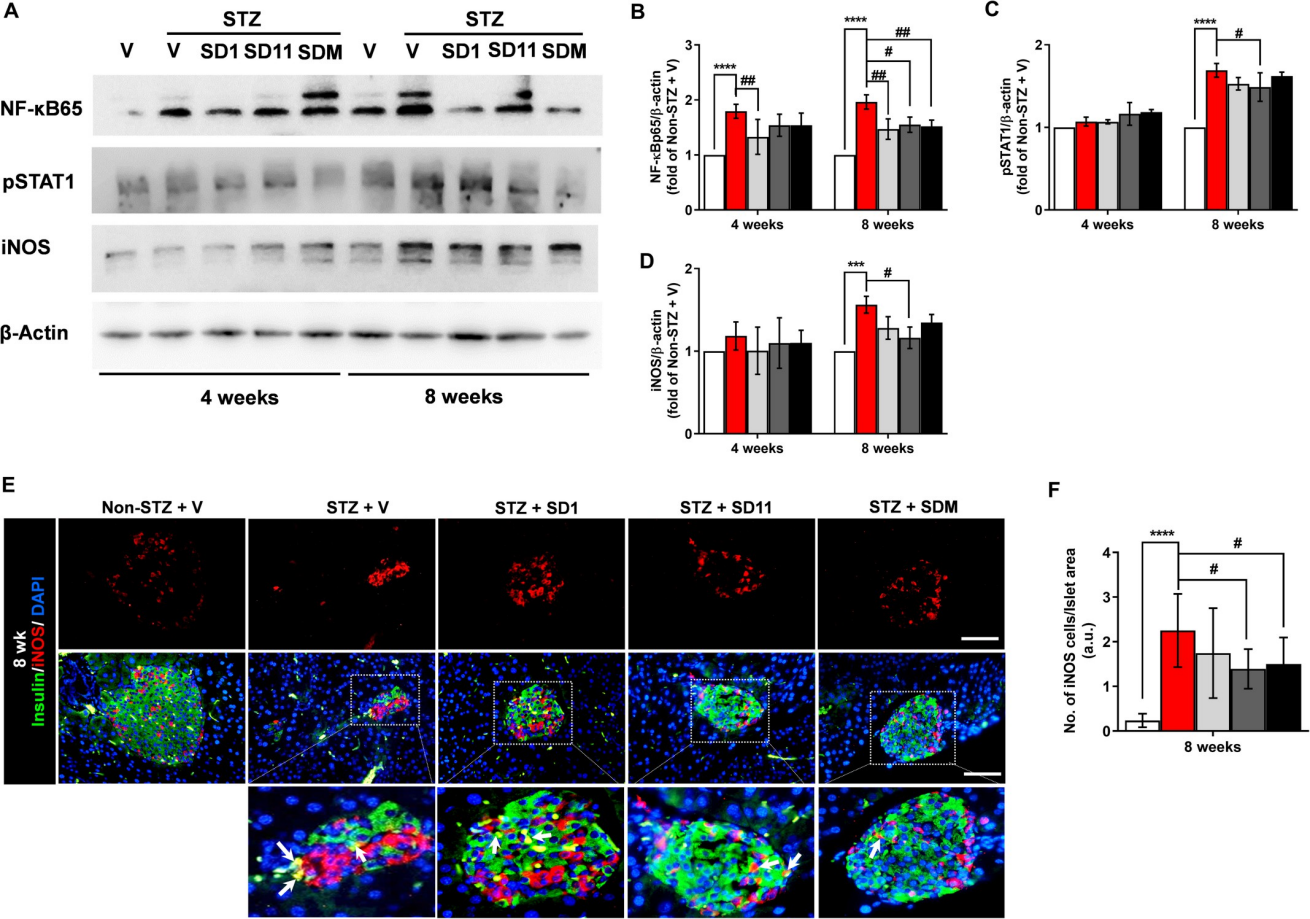
Probiotics counteract the cytokine-induced β-cell toxic signaling pathway. (A) Representative images of pancreatic protein expression of NF-κBp65, pSTAT1, and iNOS were measured by Western blotting. Densitometry quantification of (B) NF-κBp65, (C) pSTAT1, and (D) iNOS normalized to β-actin. (E) Representative immunofluorescent images of the M1 polarized macrophage marker iNOS (red), β-cell marker insulin (green) and DAPI (blue) staining in sections of formalin-fixed paraffin-embedded among experimental groups at 8 weeks. White arrows indicate localization of insulin- and iNOS-positive cells. (F) Quantification of the number of iNOS-positive cells/islet area. All images were taken at 400 magnification. Scale bars, 50 μm. Data represent the mean ± SEM. N = 3 for Western blot assay. A measurement of 50 independent islets per group (10 islets per mouse pancreas were randomly chosen from 5 animals per group) was examined for immunostaining. ****P < 0.0001, ***P < 0.001 vs. the corresponding non-STZ group; ^##^*P* < 0.01, ^#^*P* < 0.05 vs. the corresponding vehicle-treated STZ group.

STAT1 and NF-κBp65 are key transcription factors that censor the activation of cytokines; hence, they are chief regulators modifying the intracellular β-cell apoptotic pathway [3]. (Fig 6A–6D). One of the mechanisms of cytokine-induced islet β-cell damage results from the production of iNOS by classically activated M1 macrophages and even by β-cells themselves [25]; therefore, it is an important marker for M1 macrophage activation [25,26]. We then further tested their changes in the cytokine-mediated β-cell apoptotic cascade. Our results showed that although PSAT1 and iNOS levels did not change significantly at 4-weeks, a significant increase in all markers was detected in pancreatic lysates in vehicle-treated STZ mice at the 8-week time point compared to those from the non-STZ group (Fig 6A–6D). Nevertheless, each probiotic treatment effectively counteracted apoptotic regulators in a time-dependent manner. As shown in Fig 6A and 6B, we observed that NF-κBp65 is likely to play a crucial role in our mouse models and that SD1 significantly reduced NF-κBp65 production in the pancreas at both time points, while SD11 and SDM had a significant impact only at the 8-week time point compared to vehicle-treated STZ mice (Fig 6A and 6B). Furthermore, pSTAT1 production was reduced significantly by SD11 treatment for 8 weeks. (Fig 6A and 6C). Meanwhile, the suppression of iNOS was statistically significant in SD11-treated mice at the 8-week time point (Fig 6A and 6D), whereas a similar effect was not found at 4 weeks. In parallel, immunostaining with iNOS antibody showed that SD11 and SDM treatments significantly reduced the number of proinflammatory M1 macrophages (iNOS-positive staining cells) in the diabetic pancreas compared to vehicle-treated STZ mice (Fig 6E and 6F). Additionally, costaining with insulin antibody revealed that some insulin-positive cells colocalized with iNOS expression (Fig 6E, white arrows), which further supports previous studies that iNOS could also be activated in β-cells. Thus, the administration of probiotics could protect against a certain degree of β-cell destruction through the downregulation of key apoptotic execution molecules in the STZ-induced diabetic model.

## Discussion

Through extensive literature reviews, Lactobacilli are one of the most frequently used bacteria in probiotics for humans because of their various health-promoting properties, including antioxidant [27] and glucose-lowering effects [27–29] and their tendency to balance gut microbiota [30] and regulate immune function [28,31]. Indeed, *L*. *rhamnosus* GG is well studied, and its effects on human health have been examined in numerous clinical studies [31,32]. In this work, two selected probiotic strains, SD1 and SD11, have been shown to confer health benefits in clinical trial studies, as they can reduce populations of cariogenic *S*. *mutans* with no adverse consequences after long-term consumption in volunteer individuals [15–17]. Therefore, our work aimed to explore and broaden the preclinical applications of SD1 and SD11 in STZ-treated mice in the aspects primarily associated with hyperglycemic phenotype-driven pancreatic-inflammatory responses. Since the beneficial effects of probiotics are strain specific, not all probiotics will demonstrate similar effects on each signaling pathway [6]. Our data support this notion, and we found that although their ability to alleviate hyperglycemia-driven β-cell damage were similar, each probiotic strain played essential roles to different degrees in each marker tested. Based on all our observations, SD11 supplementation exhibits the most profound outcomes. A possible explanation for why the mixture of SD1 and SD11, designated SDM, did not result in a better protective property when compared to a single strain treatment of SD1 or SD11 is that the total concentration of probiotics in the SDM treatment was 10^9^ CFU/mL (not a double dose). Overall, supplementation with SD1, SD11, or SDM in STZ-injected mice resulted in the following: 1) maintenance of body weight and improved blood sugar levels, 2) improved OGTT and serum lipid index, 3) reduced intraislet immune cell infiltration and inflammation, and 4) alleviation of programmed cell death via the apoptotic pathway.

The animal model used in this study was successfully induced by multiple injections of low-dose STZ (Fig 1). All STZ-injected mice developed hyperglycemia 72 h after the last injection, and the FBG increased continuously, especially in vehicle-treated STZ mice ranging from 220–432 mg/dL, which is significantly higher than treatment with SD1 (222–320 mg/dL), SD11 (214–350 mg/dL) and SDM (222–369 mg/dL). Body weight is also an indicator of DM symptoms. The decreased body weight in diabetic mice is due to insulin deficiency, and fat and protein catabolism. The slight improvement in body weight by probiotics could be due to better glycemic control in these mice, which led to improvement in their energy intake, therefore compensating for the loss in body weight. Other hyperglycemia-associated symptoms, including polydipsia and polyphagia, gradually improved in probiotic-treated mice as well.

Abnormal elevated glucose levels are often accompanied by lipid metabolic disruption. Generally, hyperlipidemia in T1D patients is primarily associated with insulin deficiency, meaning that the enzyme hormone sensitive lipase (HSL) is not inhibited, thereby increasing the mobilization of FFAs. In addition, the catabolism of glucagon is not altered by insulin, so the net result is the release of FFAs, and serum lipid levels can double [33]. Therefore, we further examined serum TC, TG, LDL-c, and HDL-c (Table 1). Our results showed that SD11- and SDM-treated mice reduced the risk of hyperglycemia-related factors while lowering TC, TG, and LDL-c and increasing HDL-c in serum. On the other hand, probiotic SD1 exhibited protective effects only at 4 weeks, and its protection did not last until the end of the experiment. Although a significant difference was not detected, all probiotic-treated mice tended to reduce FFA in serum after 8 weeks of treatment. Moreover, an improved glycemic index alleviates relevant parameters, including OGTT, fasting insulin levels, and insulin resistance in HOMA-IR. Our observation is further in agreement with previous studies [6,34]. Thus, we speculate that the hypolipidemic effect of our probiotics could potentially be attributed to the higher circulating insulin, which increases glucose uptake and inhibits HSL. However, the underlying mechanism by which probiotics decrease insulin resistance or increase sensitivity to insulin for glucose uptake requires further study. Taken together, these results explained that controlling blood glucose levels by probiotics played an important role in improving lipid profiles in STZ-injected mice.

During T1D pathogenesis, autoimmune-induced cell destruction contributes to the loss of β-cell mass [3]; hence, a strategy to ameliorate β-cell death is essential. Our observation by both H&E and immunofluorescent examinations revealed that after 8 weeks of probiotic intervention, the arrangement of islet cells gradually became regular, and the number of islet and β-cells were higher than those of vehicle-treated STZ mice (Fig 2). Since macrophages act as innate immune effector cells and manifest inflammatory responses by recruiting other immune cells, such as lymphocytes, to the pancreas, we then took a closer look at the infiltration of macrophages expressing the antigen F4/80^+^, a marker of macrophages, and it was found that F4/80^+^ accumulated in the pancreatic islets of STZ-induced diabetic mice [35]. Upon enhanced macrophage recruitment, CD4^+^ and CD8^+^ T cells are activated and lead to insulitis, where cytotoxic T cells aggravate β-cell death, as shown by the increased number of CD4^+^ and CD8^+^ T cells correlated with the number of F4/80^+^ positively stained cells in the vehicle-treated STZ pancreas. However, the intrainfiltration of these innate and adaptive immune cells was lower in the probiotic-treated pancreas, especially after the treatment with SD1 and SD11 (Fig 3). These data suggest that probiotics could improve the intraislet environment and preserve islet integrity and β-cell mass, allowing them to function properly. This phenomenon clearly provides an explanation for why glycemic and insulin indices were better in probiotic-treated mice than in untreated STZ mice. As a subsequent event after CD4^+^ and CD8^+^ T cells are activated, the three most important proinflammatory cytokines, IL-1β, TNF-α, and IFN-γ, are released [1,3]. The results in Fig 4 show that all probiotic-treated groups had lower levels of IL-1β, TNF-α, and IFN-γ protein expression in the pancreas and higher levels of IL-10, which is an essential element in maintaining self-immune tolerance, than untreated STZ mice. This result is consistent with other studies demonstrating that increased IL-10 inhibits the secretion of the proinflammatory cytokines TNF-α, IL-1β, IL-2 and IL-6 after treatment with *L*. *kefiranofaciens* in STZ-induced diabetic mice for 8 weeks [36]. However, future studies are required to determine the number of circulating lymphocytes and cytokines and the expression of regulatory T cells to clarify the underlying mechanisms.

Our study further demonstrated, by Western blot and immunofluorescence analysis, that supplementation with SD1, SD11, or SDM resulted in significant downregulation of cleaved-caspase 3, caspase 9, caspase 8, and Bax proteins and upregulation of Bcl2 protein in the pancreas (Fig 5). As a critical regulator of cell apoptosis, Bcl2 family proteins consist of both proapoptotic and antiapoptotic members. Among these markers, Bax possesses a pro-apoptotic effect by inducing the release of cytochrome C from mitochondria, leading to caspase activation cascades and cell apoptosis. In contrast, Bcl2 plays an anti-apoptotic role either by requesting pro-caspases or by preventing the release of cytochrome C [24]. Consistent with the modulating effect of SD1 and SD11 on the apoptotic cascade, supplementation with SD1 and SD11 markedly decreased the levels of cleaved-caspase 3, caspase 9, and caspase 8. These data highlight that regulation of Bcl2 protein and subsequent inhibition of caspase activation could play a critical role in cytokine-induced β-cell death of SD1 and SD11 in the STZ-induced pancreas.

Finally, we explored the molecular mechanisms underlying these anti-inflammatory and anti-apoptotic properties of SD1 and SD11 in the pancreas of STZ mice. The NF-κB pathway is a key modulator of inflammatory mediators such as IL-1β that are common determinants of β-cell survival, but when it is abnormally activated, it leads to β-cell death by upregulating an important player, iNOS [9]. Moreover, recent studies have shown that proinflammatory M1 macrophages preferentially induce iNOS expression, resulting in nitric oxide (NO) production and a T-helper 1 (Th1) CD4^+^ T-cell response [25,26]. Our immunofluorescence results further support that SD11 and SDM could alleviate the number of infiltrating proinflammatory M1 macrophages and β-cells expressing iNOS (Fig 6, white arrows). We conclude that both macrophages and β-cells express iNOS and that iNOS production could be activated by IL-1β and IFN-γ released from macrophages and T cells in the diabetic pancreas. This finding implies that iNOS could be suicidal from β-cells themselves as well as homicidal from macrophages. Therefore, the suppression of iNOS expression by SD11 contributes to increased β-cell viability and eventually improves the secretory response to glucose. Additionally, evidence reports that in T1D, once the proinflammatory cytokine IFN-γ is released, it promotes the recruitment and phosphorylation of STAT1. After being phosphorylated, STAT1 homodimerizes and migrates to the nucleus to initiate the transcription of apoptosis-inducing genes, which eventually induce β-cell death through the apoptotic cascade [24,34]. All these disturbances were significantly reversed by SD1 and SD11 at the 8-week time point, as presented in the present study (Fig 6). The downregulation of the NF-κB pathway not only relieves β-cell death but may also contribute to reduced β-cell sensitivity to cytokines and reduced cytokine secretion, further reducing T-cell infiltration into the islets. These data suggest that SD1 and SD11 could reduce the severity of cytokine-induced β-cell apoptosis in STZ pancreases.

Research documents indicate the strong link between probiotics and the gut microbiome, but how they interact is rather complex due to several factors, including age, genetics, diet, location, physiological and psychological stresses [5]. Considering the limitations of our present work, there is a need for additional research in the future to validate the preclinical and clinical applications of SD1 and SD11. For example, gut microbiota composition and secreted soluble factors and metabolites such as SCFAs should be clarified. This set of data will enable us to gain more understanding and meaningful insights into the effectiveness of SD1 and SD11. Nonobese diabetic (NOD) mice can also provide more precise mechanisms regarding the immune function of probiotics.

## Conclusion

Here, we reported for the first time that probiotic SD1 and SD11 supplements contribute to not only alleviation of metabolic dysfunction but also modulation of subsequent glucotoxicity by relieving the insulin index and pancreatic inflammation at least partly by reducing the intraislet immune cell infiltration and promoting the production of a more suppressive and/or regulatory cytokine, IL-10, while suppressing proinflammatory cytokines, IL1-β, TNF-α and IFN-γ, eventually preventing the upregulation of pancreatic β-cell apoptotic modulators. Although it is unlikely that probiotics alone can act as a therapy for replacing modern medicine, our findings support that SD1 and SD11 could be used concomitantly to produce the safest and most effective clinical outcome as an intervention for the hyperglycemia-induced inflammatory response in T1D.

## Supporting information

S1 File Supplemental raw data. (XLSX)

S2 File The ARRIVE guidelines 2.0: Author checklist. (PDF)

S1 Raw images Original unedited blot results. (PDF)
