# Supplementary material for: Probiotics of Lacticaseibacillus paracasei SD1 and Lacticaseibacillus rhamnosus SD11 attenuate inflammation and β-cell death in streptozotocin-induced type 1 diabetic mice
Source: PLoS One. 2023 Apr 11;18(4):e0284303. doi: 10.1371/journal.pone.0284303 (PMC10089358; doi:10.1371/journal.pone.0284303)

S1 Fig. Unedited images of Fig 4. M: Protein marker.

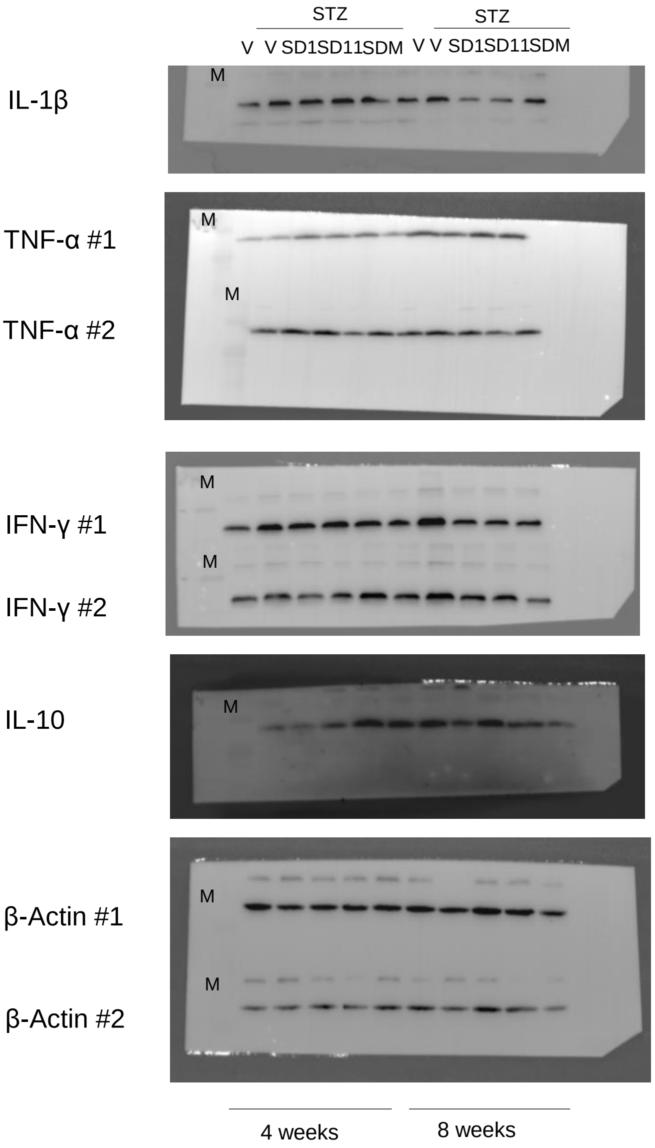

S1 Fig. Unedited images of Fig 5. M: Protein marker.

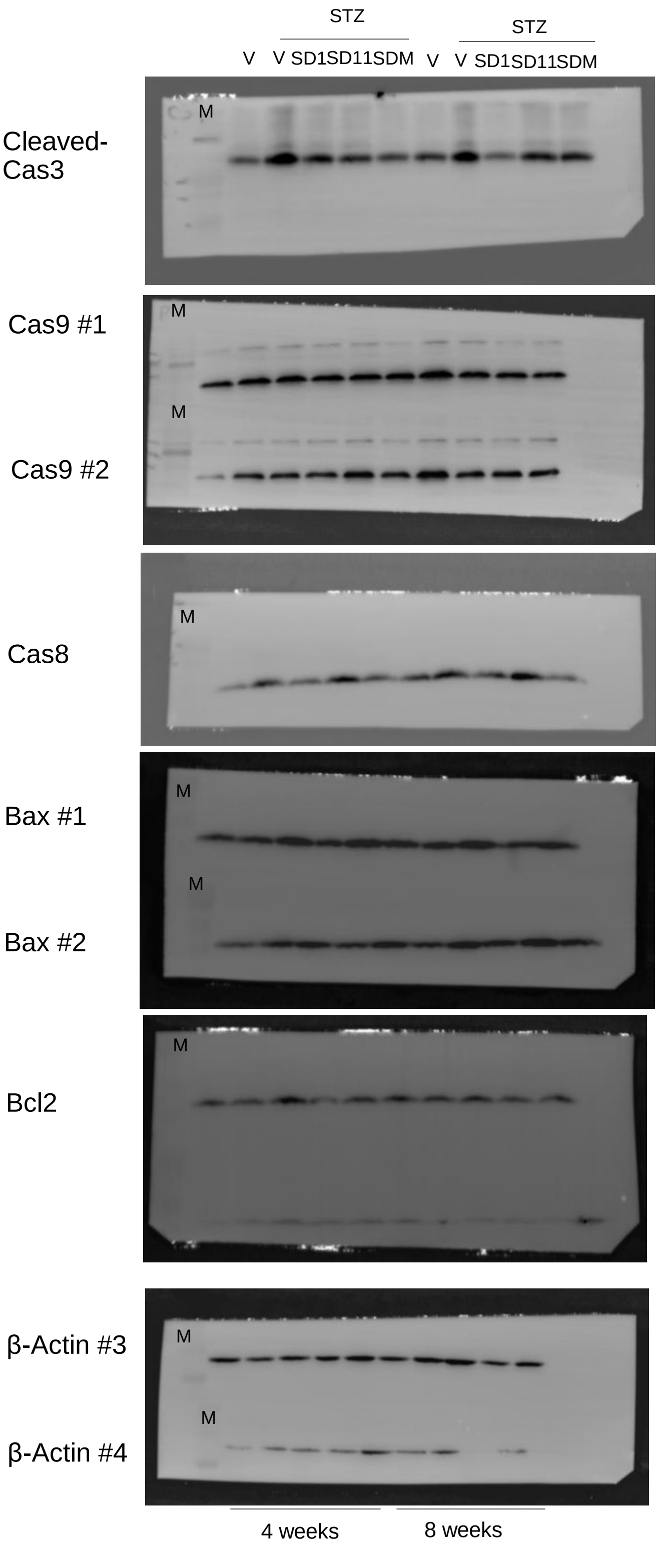

S1 Fig. Unedited images of Fig 6. M: Protein marker.

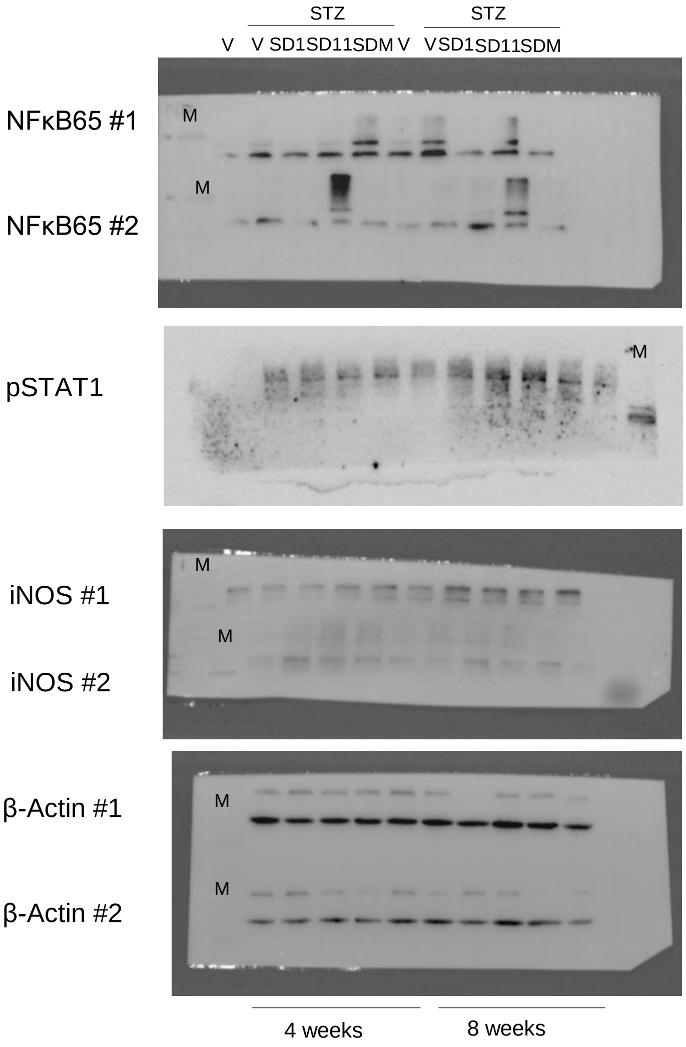

Supplement: S1 Raw images — (PDF) [file pone.0284303.s003.pdf]
